# Bioactive Ingredients and Medicinal Values of *Grifola frondosa* (Maitake)

**DOI:** 10.3390/foods10010095

**Published:** 2021-01-05

**Authors:** Jian-Yong Wu, Ka-Chai Siu, Ping Geng

**Affiliations:** Food Safety and Technology Research Center, Department of Applied Biology & Chemical Technology, The Hong Kong Polytechnic University, Hung Hom, Kowloon, Hong Kong; jian-yong.wu@polyu.edu.hk (J.-Y.W.); ka-chai.siu@polyu.edu.hk (K.-C.S.)

**Keywords:** *Grifola frondosa*, maitake mushroom, polysaccharide, molecular structures, functional foods

## Abstract

*Grifola frondosa* (*G. frondosa*), generally known as hen-of-the-woods or maitake in Japanese and hui-shu-hua in Chinese, is an edible mushroom with both nutritional and medicinal properties. This review provides an up-to-date and comprehensive summary of research findings on its bioactive constituents, potential health benefits and major structural characteristics. Since the discovery of the D-fraction more than three decades ago, many other polysaccharides, including β-glucans and heteroglycans, have been extracted from the *G. frondosa* fruiting body and fungal mycelium, which have shown significant antitumor and immunomodulatory activities. Another class of bioactive macromolecules in *G. frondosa* is composed of proteins and glycoproteins, which have shown antitumor, immunomodulation, antioxidant and other activities. A number of small organic molecules such as sterols and phenolic compounds have also been isolated from the fungus and have shown various bioactivities. It can be concluded that the *G. frondosa* mushroom provides a diverse array of bioactive molecules that are potentially valuable for nutraceutical and pharmaceutical applications. More investigation is needed to establish the structure–bioactivity relationship of *G. frondosa* and to elucidate the mechanisms of action behind its various bioactive and pharmacological effects.

## 1. Introduction

*Grifola frondosa* (*G. frondosa*) is a Basidiomycetes fungus that belongs to the family of Grifolaceae and the order of Polyporales. In Japan its edible fruiting body is known as maitake. In Japanese, *mai* means dance and *take* means mushroom. *G. frondosa* is known as “hui-shu-hua” (grey tree flower) in Chinese, possibly due to its appearance. *G. frondosa* grows around the stumps of broadleaf trees or trunks and is edible when young. The environment of the northeastern part of Japan is suitable for the growth of *G. frondosa*. The temperate forests in eastern North America, Europe and Asia are also ideal for its growth. Meanwhile, it is a common mushroom in the Unites States and Canada, known as sheep’s head, king of mushrooms, hen-of-the-woods, and cloud mushroom [1].

**Figure 1:**
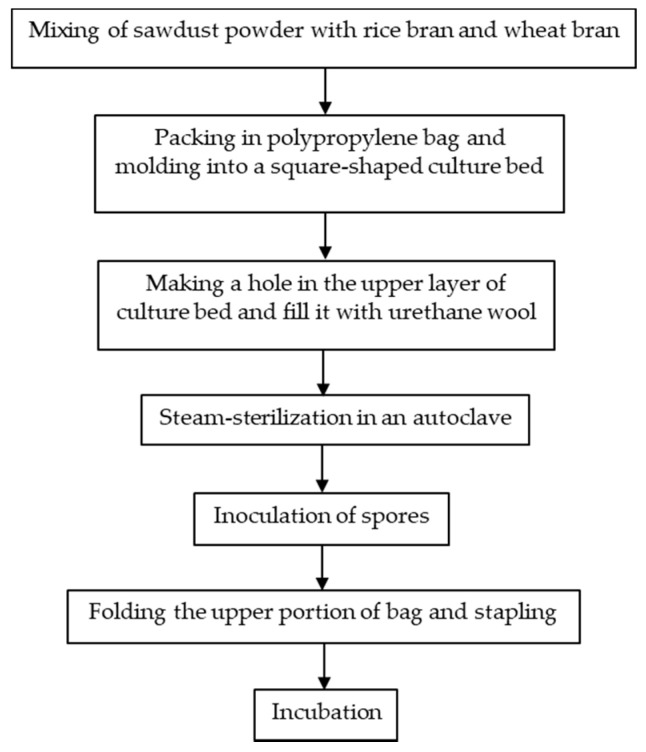
A typical bag culture procedure for the *G. frondosa* fruiting body.

**Figure 2:**
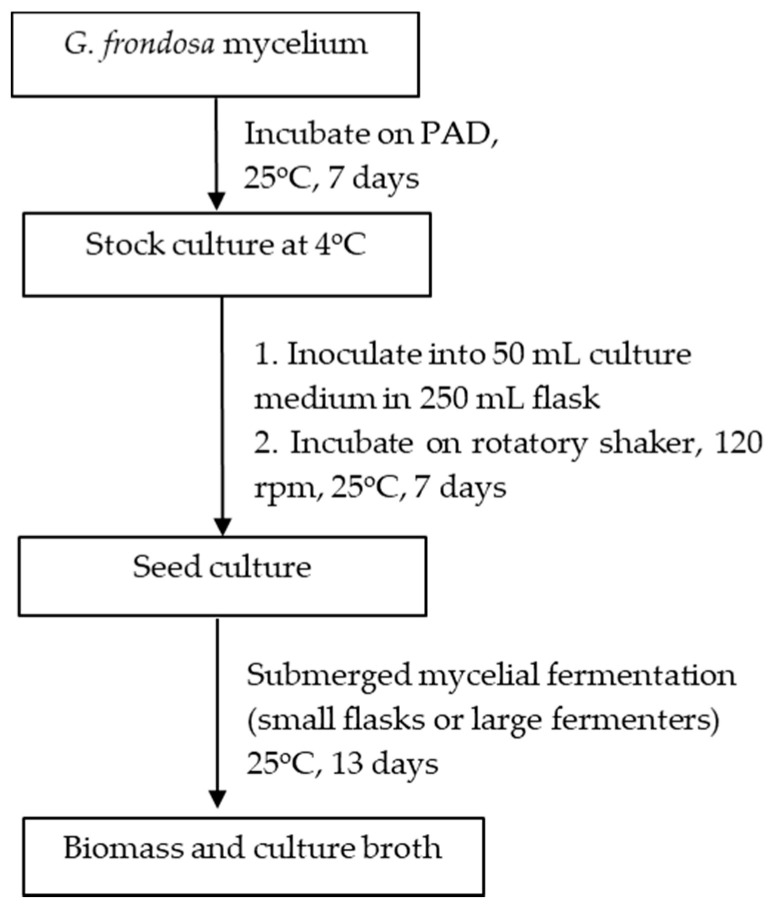
Submerged culture fermentation of the *G. frondosa* mycelium adapted from [4].

Japan was one of the countries that first started the artificial cultivation of *G. frondosa* in the mid-1980s. There are in general three methods for the artificial cultivation of the *G. frondosa* fruiting body, they are bottle culture, bag culture and outdoor bed culture. Bag culture is the most popular cultivation method in Japan [2] because of its advantages such as the low cost of plastic bags, small space requirements and easily-controlled indoor environment. Bag culture can achieve higher yields of mature *G. frondosa* mushrooms than bottle culture and requires a shorter cultivation time than outdoor bed cultures. As shown in Figure 1 [2], the major steps of bag cultivation include substrate preparation, substrate sterilization, mycelium inoculation and incubation. In addition to the fruiting body, there is also an increasing demand for *G. frondosa*’s mycelium and its bioactive metabolites. Solid-state fermentation (SSF) [3] and submerged fermentation [4] are two common methods of mycelium cultivation. A common substrate for SSF is sawdust supplemented with rice bran or wheat bran [5]. Submerged or liquid fermentation is usually more efficient, providing a higher mycelial productivity in a shorter time, requiring smaller plant space and allowing for more effective product quality control [6]. A typical submerged fermentation process is presented in Figure 2.

*G. frondosa* is edible and is regarded as a healthy food because it is a good source of protein, carbohydrates, dietary fiber [7,8,9,10,11,12,13], vitamin D_2_ (ergocalciferol) [13,14,15] and minerals (K, P, Na, Ca, Mg) [7,9,12,15,16], with low fat content and caloric value [15]. *G. frondosa* is delicious, with a sweet and umami taste, which is mainly attributed to its high trehalose, glutamic and aspartic amino acid and 5′-nucleotide content [10,11,13,17]. Due to its delicious and special taste, *G. frondosa* is not only used as a food ingredient, but also as a food-flavoring substance in dried powder form. Apart from its high nutraceutical value, *G. fondosa* is reported to possess a wide range of pharmacological effects. *G. frondosa* was first discovered to have antitumor activity in the 1980s from hot water extracts of the *G. frondosa* fruiting body [16,17]. The major bioactive components were found to be β-glucans [17,18,19,20]. The D-fraction, a β-glucan complex with about 30% protein, was first discovered by Nanba’s group in the late 1980s [21]. Since then, the D-fraction has been widely studied and gradually developed into commercially available complementary medicines and healthcare products. In addition to the D-fraction, there are many other bioactive polysaccharide fractions that are obtained from *G. frondosa*, such as the MD-fraction [22], X-fraction [23], Grifolan [24], MZ-fraction [25] and MT-α-glucan [26]. The different polysaccharide fractions isolated from *G. frondosa* possess various bioactive effects such as immunomodulation [24], antitumor [25], antivirus [27], antidiabetic [26] and anti-inflammation [28]. In recent years, an increasing number of studies have attributed or linked the health and therapeutic effects of *G. frondosa* polysaccharides to their capacity for modifying gut microbiota, microorganisms that play an important role in human health and diseases. In particular, gut microbiota play a role in maintaining immune homeostasis, which may have a connection to the antitumor effects of polysaccharides [29]. The regulation of gut microbiota composition by *G. frondosa* polysaccharides has also been suggested to contribute to the treatment of metabolic disorders such as non-alcoholic fatty liver disease (NAFLD) [30] and diabetes [31], indicating their potential for preventing or treating hyperglycemia and hyperlipidemia. Apart from polysaccharides, other molecular fractions isolated from *G. frondosa* fruiting bodies or mycelial biomass have shown promising medicinal values as well. For instance, the protein components of *G. frondosa*, including glycoprotein, have shown anti-tumor [32], immune-enhancing [33], anti-diabetic, anti-hypertensive, anti-hyperlipidemic [34] and anti-viral effects [35]. Moreover, other small biomolecules in *G. frondosa* have been found to possess health benefits such as anti-inflammation [36], hypoglycemia [37], antitumor [38] and antioxidation [39].

This review gives an up-to-date and comprehensive summary and assessment of the basic composition and bioactive components of *G. frondosa*, with an overview of their structural characteristics and bioactivities. It has two major parts. The first part covers the composition and nutritional effects of the *G. frondosa* fungus and the second part focuses on its medicinal properties, involving major bioactive molecules, their structural characteristics and bioactivities. Given that there are relatively few reviews in the literature which provide an overall picture in terms of both the nutritional and medicinal values of *G. frondosa*, this review may provide a useful and up-to-date reference for further research on the constituents, properties and functions of *G. frondosa* and for development and commercial applications in the form of new functional foods and therapeutic products.

## 2. Chemical and Nutritional Compositions

### 2.1. Proximate Composition

Generally, proximate composition is determined by the methods suggested by the Association of Official Analytical Chemists (AOAC). The total carbohydrate content can be calculated by subtracting the percentages of ash, crude fat and protein [7,40]. For the determination of crude protein, the nitrogen conversion factor is 4.38 instead of the usual 6.25, due to the large amount of chitin that is usually contained within the fungus, a component that may interfere with the correct calculation of the result of total nitrogen [41].

As shown in Table 1, *G. frondosa* is made up of around 83–96% moisture and 4–17% dry matter in its fresh fruiting body [7,8,9,10,11,12,13] and mycelium [11,17,42], indicating the watery texture of *G. frondosa*. Carbohydrates and protein are the major constituents contributing to the dry weight of *G. frondosa*, taking up around 70–80% and 13–21%, respectively, of the fruiting body. Based on the average values of component percentage, it could be found that the mycelium of *G. frondosa* has a similar moisture content, a lower content of carbohydrate and crude ash and a higher content of crude fat and protein, compared with the fruiting body of *G. frondosa*.

According to the research findings of Kurasawa and coworkers [8], the composition of the *G. frondosa* fruiting body resembles that of normal cultivated mushrooms. It is worth mentioning that the crude fat content of the *G. frondosa* fruiting body is generally lower than the average crude fat content in cultivated mushrooms (4.3%), and the amounts of protein and carbohydrates are slightly higher than the average of other mushrooms (17.2% and 70.3%), indicating the excellent nutritional values of *G. frondosa.*

### 2.2. Soluble Sugar Content

The content of soluble sugar within *G. frondosa* is mostly determined by the method described in the research work of Ajlouni and coworkers [42]. As shown in Table 2, the total sugar content in *G. frondosa* is higher in the fruiting body (90–190 mg/g) than in the mycelium (70–90 mg/g). The content of total sugar in the fruiting body of *G. frondosa* is also superior to some edible mushrooms such as *Lactarius glaucescens* and *Craterellus odoratus* [45], which may be one of the reasons for the good taste of *G. frondosa*. Table 2 also shows variations in both total soluble sugar content and individual sugar content among different *G. frondosa* samples, which may be attributed to factors such as cultivation period and cultivation environment. Trehalose, a disaccharide that comprises two molecules of glucose, is the major sugar component of both the fruiting body and mycelia of *G. frondosa* [9,10,11,43,44]. Compared with the amount in the mycelium (40–60 mg/g), the fruiting body contains more trehalose than the mycelium, around 50–160 mg/g in dry weight. In addition to trehalose, the fruiting body also contains glucose and mannitol, whereas the mycelium has glucose and mannitol, together with arabitol and fructose.

### 2.3. Free Amino Acid Content

The content of free amino acids in *G. frondosa* was quantitatively measured by the method described in the research work of Mau and coworkers using HPLC [10,44]. Results regarding the free amino acid content of *G. frondosa* are exhibited in Table 3. The total free amino acid content in the fruiting body of *G. frondosa* is around 15–60 mg/g in dry weight, which is higher than that in many other edible mushrooms, such as *Dictyophora indusiata* and *Tricholoma giganteum* [10]. The mycelium of *G. frondosa* contains a relatively higher total free amino acid content in comparison with the fruiting body. There is also a great variety of amino acids in *G. frondosa*. There are around eighteen kinds of free amino acids, including essential amino acids such as L-histidine and L-methionine, in both the fruiting body and the mycelium of *G. frondosa*, indicating that *G. frondosa* is an excellent source of amino acids.

However, large variations exist in the amount of each amino acid in *G. frondosa.* For instance, Mau et al. (2001) and Tsai et al. (2006) found that threonine was the major free amino acid in *G. frondosa* [10,44], whereas Huang and coworkers found that histidine and glutamic acid were the major free amino acids in the fruiting body (>12 mg/g) and lysine, aspartic acid and tyrosine were the major free amino acids in the mycelium (>17 mg/g) [11]. Huang and coworkers also obtained a larger amount of free amino acid from *G. frondosa* than other groups in both the fruiting body and the mycelium, a result that might be due to the different sources of the fruiting body and the preparation methods of the mycelium by the different research groups. The choice in cultivation substrate was also found to affect the variety and amount of amino acids in *G. frondosa*. As shown in Table 3, *G. frondosa* fruiting bodies cultivated in sawdust and log substrates have different amounts of each amino acid [13], although the total amino acid content was similar. Moreover, GABA (γ-aminobutyric acid), a biologically active compound which is related to the therapeutic effect of *G. frondosa*, is mainly detected in the mycelium but not in the fruiting body [11] (Table 3).

## 3. Bioactive Ingredients

### 3.1. Polysaccharides

**Figure 3:**
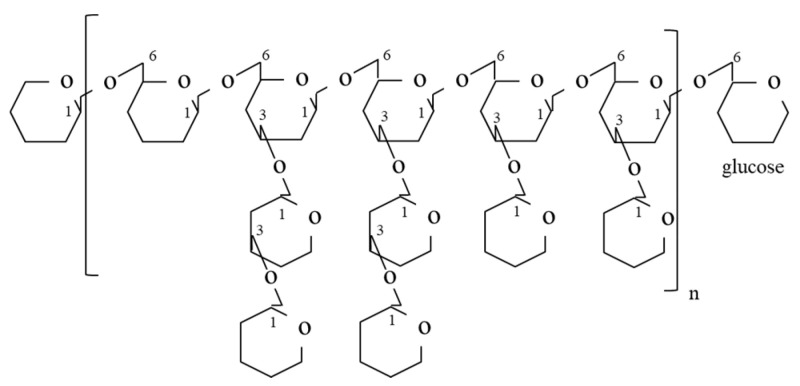
Typical structure of the D-fraction with (1→6)-glucan having a (1→3)-branched chain [49].

**Figure 4:**
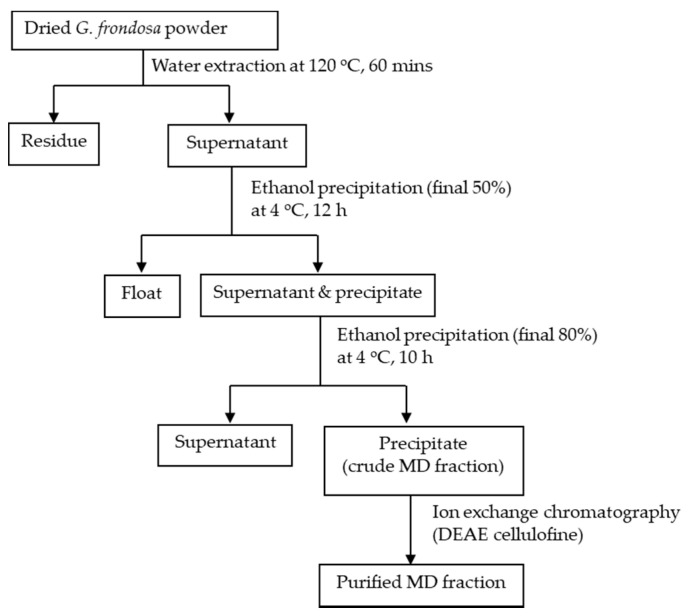
A typical extraction method of the MD fraction from *G. frondosa* adapted from [50].

In the past 30 years, over 47 bioactive polysaccharide fractions have been isolated and purified from the fruiting body, mycelium and cultured medium of *G. frondosa* using different extraction methods. As previously reported, *G. frondosa* contains 3.8% water-soluble polysaccharides on a dry weight basis, of which 13.2% was (1→3, 1→6)-β-D-glucan [46], and others include heteroglycan or the heteroglycan/protein complex [47]. Among these bioactive polysaccharide fractions, the D-fraction and the MD-fraction (purified D-fraction) [22], which are regarded as the most important bioactive polysaccharides, have been officially used as antitumor, anticancer and immunomodulatory agents [48]. Through structural characterization, Nanba and Kubo discovered the complex structure of the β-D-glucan in the D-fraction. Unlike other mushroom-derived β-glucans that contained a (1→3) main chain with (1→6) branches only, the β-D-glucan in D-fraction possessed both a (1→6) main chain with (1→3) branches and a (1→3) main chain with (1→6) branches [22]. The high molecular weight of the D-fraction was considered a factor contributing to its strong immunomodulatory effects [22]. Figure 3 shows a typical structure of the D-fraction. The D-fraction and the MD-fraction could be extracted and fractionated from both the mycelium and fruiting body of *G. frondosa*. Figure 4 is a general flowchart for the extraction and purification of the MD-fraction as reported by Nanba and coworkers. The MD fraction is typically extracted from the dried *G. frondosa* powder with boiling water and then isolated by ethanol precipitation. The precipitate (crude MD fraction) is further fractionated through column chromatography, including ion exchange and gel permeation chromatography, to obtain the purified MD fraction.

Apart from the D-fraction and the MD-fraction, other polysaccharide fractions have also been derived from *G. frondosa*, with hot water being the most commonly used extraction solvent. Ultrasound [51] and other solvents such as hot sodium hydroxide [18] and citrate buffer [24] have also been utilized. Table 4 summarizes the chemical properties, sources and extraction solvents of representative bioactive polysaccharide fractions isolated from *G. frondosa*. The chemical properties listed include molecular weight, structure information, as well as monosaccharide compositions. For instance, Kubo et al. extracted the X-fraction from the fruiting body of *G. frondosa*, which was a (1→6)-β-glucan with (1→4)-α branches. The X-fraction showed anti-diabetic activity, which was found to be directly associated with insulin receptors [23]. Masuda et al. separated the MZ-fraction from *G. frondosa*, which had similar structure to the MD-fraction but with a much smaller molecular weight, of around 20,000 Da (the molecular weight of MD-fraction was around 1–2 million Da). The MZ-fraction showed immunomodulatory effects in vitro and antitumor activity in vivo [25]. Moreover, Grifolan (GRN), a gel-forming (1→6)-branched (1→3)-β-D-glucan, was found in *G. frondosa* with immunomodulatory effects [24]. α-D-glucan could also be extracted by hot water from *G. frondosa*. Instead of antitumor activity, hypoglycemic, hypolipidemic, antioxidative and immunomodulatory effects were discovered [26,52,53,54]. In recent years, Liu’s group from China has isolated various polysaccharides from *G. frondosa* with the names of GFP-A [55], LMw-GFP [51] and GFAP [56], all of which exhibited promising anti-tumor activities. Due to variations in external factors such as the fungal source and extraction temperature, the properties of polysaccharide fractions from different sources may vary significantly.

### 3.2. Proteins and Peptides

Several kinds of bioactive proteins and peptides have been isolated from *G. frondosa* with notable health benefits and medicinal values, although studies on these aspects are fewer than those on the bioactive polysaccharide fractions. Table 5 summarizes some typical bioactive proteins and peptides isolated from *G. frondosa*. As shown in the table, these bioactive proteins/peptides were extracted mainly from the *G. frondosa* fruiting body, with average molecular weights around 20–88 kDa.

The N-acetylgalactosamine-specific lectin GFL isolated from the *G. frondosa* fruiting body exhibited cytotoxicity against HeLa cells [71]. The protein designated GFAHP showed a significant anti-herpes simplex virus (HSV) effect, as reported by Gu and coworkers [35]. Glycoprotein is another type of bioactive protein in *G. frondosa*. Cui and coworkers isolated the glycoprotein (containing 6.2% carbohydrates) from cultured mycelia of *G. frondosa* and demonstrated its anti-tumor activity [32]. Zhuang and coworkers patented a bioactive glycoprotein from *G. frondosa*, which showed obvious anti-hypertensive, anti-obesity, anti-hyperlipidemic and anti-diabetic effects [34]. In addition to the glycoprotein, Chan et al. found that chemical phosphorylation of *G. frondosa* polysaccharide-peptides could remarkably enhance both tumor inhibition in vitro and adjuvant effects in vivo. Meanwhile, modified and unmodified MPSP both showed an insignificant effect on normal cells, indicating their potential application for anticancer therapy without significant side effects [72].

### 3.3. Other Bioactive Molecules

Apart from the macromolecular components, such as polysaccharides and proteins/peptides, bioactive small molecules have also been discovered in *G. frondosa*. Table 6 lists some bioactive small molecules in *G. frondosa* from representative research studies. The major small molecules discovered with bioactivities mainly include fatty acids, ergosterols, flavonoids, alkaloids, ascorbic acid and tocopherol.

As reported by Zhang and coworkers, fatty acids and three compounds, namely ergosterol (1), ergostra-4,6,8(14),22-tetraen-3-one (2) and 1-oleoyl-2-linoleoyl-3-palmitoylglycerol (3), were extracted from the cultured mycelia of *G. frondosa* by hexane. The fatty acid fraction, together with all three compounds, exhibited cyclooxygenase (COX) enzyme inhibitory and anti-oxidant activities [73]. He and coworkers extracted a furanone named grifolaone A from *G. frondosa*, which showed specific antifungal activity against the opportunistic human pathogen of *Pseudallescheria boydii* and some plant pathogens [74]. Han and Cui isolated agaricoglycerides (AGF) from the fermented mycelium of *G. frondosa*. Their study suggested a promising possibility of using AGF as medicine for inflammatory pain with 500 mg/kg as the optimal dosage [36]. Chen et al. extracted three fractions, GF-1 to GF-3, from *G. frondosa* and discovered the inhibitory effect of GF-3 against the proliferation of human tumor cells and α-glucosidase. The major bioactive compounds in GF-3 were detected to be alkaloids (first found in *G. frondosa*), ergosterols and a new compound named pyrrolefronine. Since α-glucosidase was involved in the hydrolysis of starch into disaccharide sugars, inhibition of its activity indicated possible reduction of blood glucose level [37]. *o*-orsellinaldehyde, which showed obvious tumoricidal activity, especially selective cytotoxic effect against Hep 3B cells through apoptosis, was also extracted from a submerged culture of *G. frondosa* by Lin and Liu [38]. In addition, other bioactive molecules in *G. frondosa*, such as polyphenolics, α-tocopherol, ascorbic acid and flavonoids, were reported to have anti-oxidant properties [39].

## 4. Biological Activities and Medicinal Properties

### 4.1. Antitumor Effects

Previous studies over the past 30 years have strongly suggested that there are three possible ways by which *G. frondosa* exerts its anticancer effect—they are protection of healthy cells, prevention of tumor metastasis and inhibition of tumor growth. In other words, *G. frondosa* can fight against tumors both directly and indirectly via enhancement of the immune system. This section will mainly focus on the direct antitumor function of *G. frondosa*, whereas the immunomodulatory effect will be discussed in the next section.

The antitumor activity of *G. frondosa* was first reported by Miyazaki et al. in 1982 [16], followed by their further study on the chemical structure of glucans extracted from the *G. frondosa* fruiting body and their antitumor activity against Sarcoma 180 tumors in Institute of Cancer Research (ICR) mice [17]. Nanba’s group obtained different polysaccharide fractions and reported the D-fraction for the first time in 1988 [21]. Unlike many other antitumor polysaccharides derived from *Basidiomycetes*, which may become ineffective if administered orally, the D-fraction exhibited promising prospects, because it could be administered orally, intravenously and intraperitoneally [21]. Nanba and Kubo conducted a nonrandomized clinical study to assess the effects of the D-fraction from *G. frondosa* on 165 advanced cancer patients who received the D-fraction as crude powder tablets alone or in addition to chemotherapy. Results showed that *G. frondosa* was effective against breast, liver and lung cancers, but less effective against leukemia, stomach and bone cancers [22]. Further studies conducted by Alonso et al. demonstrated that the D-fraction was able to function on mammary tumor cells directly through the modulation of different cellular processes during cancer development [48]. Zhao and coworkers found that a combination of the D-fraction (0.2 mg/mL) and vitamin C (0.3 mmol/L) resulted in a 70% reduction in the viability of human hepatocarcinoma SMMC-7721 cells [76]. Further purification of the D-fraction yielded the MD-fraction, which, as described in the patent of Nanba and Kubo, showed even better results than the D-fraction in terms of the inhibitory effect on mouse tumor growth [22]. In addition to intraperitoneal injection in Nanba and Kubo’s test, the MD-fraction has also been demonstrated to inhibit tumor growth in mice via oral administration [77]. Both the D-fraction and the MD-fraction were proven safe, with low or no toxicity.

Apart from the D and MD-fractions, other polysaccharide fractions have also exhibited anti-tumor activity. As reported by Bie et al. [65], the polysaccharide GFP-A, isolated from *G. frondosa*, was able to inhibit the proliferation of human colon cancer HT-29 cells in vitro, with 180 μg/mL as the most effective concentration. Li and Liu reported that the polysaccharide fraction GFP-4, extracted from *G. frondosa*, showed an inhibitory effect on human lung cancer cells at 4 °C. The inhibitory effect became lower after heat treatment at over 30 °C due to structural changes [78]. Alonso and coworkers explained that the polysaccharides in *G. frondosa* could regulate gene expression involved in the apoptosis of breast cancer cells so that cell proliferation was inhibited and the cell cycle was blocked [79].

In addition to polysaccharide fractions, the ergosterol derivatives from non-polar extracts of *G. frondosa* were also found to have anti-proliferative effects on human tumor cells [37]. Moreover, the *ο*-orsellinaldehyde component of submerged cultures of *G. frondosa* exhibited tumoricidal activity against Hep 3B cells via apoptosis [38]. Some glycoproteins isolated from *G. frondosa*, such as GFL and GFG-3a, exhibited antitumor effects as well due to their anti-proliferative activity against cancer cells [32,71]. Table 7 summarizes the testing methods and potency of antitumor activity based on reported studies on *G. frondosa*. The testing methods for in vivo studies mainly include microscopy observation and assessment of the inhibition rate by measuring tumor weight, whereas for in vitro studies, the MTT assay is commonly used to determine cells’ viability. It is worth mentioning that *G. frondosa* was able to achieve an inhibition ratio of over 90% for the treatment of MM46 liver carcinoma, BEL 7402 cells and TMK-1 gastric cancer cells.

### 4.2. Immunomodulation

Immunomodulation is the most well-known effect of *G. frondosa* components and has been confirmed by many studies. These immunomodulatory components have been shown to enhance the actions of macrophages and many other immune-related cells, such as cytotoxic T-cells and natural killer (NK) cells [99]. Furthermore, *G. frondosa* components could increase the secretion of cytokines, which are signaling molecules, including interferons (IFN), interleukins (IL), tumor necrosis factors (TNF) and lymphokines with antiproliferative activity, causing apoptosis and differentiation in tumor cells, thus further increasing the efficiency of immune-related cells.

Polysaccharides have been recognized as the major immunomodulating components of *G. frondosa*. The D-fraction, in addition to its direct antitumor effect as mentioned previously, is also a major polysaccharide fraction of *G. frondosa* with significant immunomodulatory activity. Kodama and coworkers suggested that the D-fraction could activate NK cells through upregulating their expression of TNF-α and interferon-gamma (IFN-γ) proteins. Meanwhile, the D-fraction were also able increase macrophage-derived IL-12, which further activated NK cells, implying that the D-fraction could provide long-term tumor-suppressive effects [49,82]. Further investigation by Kodama et al. found that the application of the D-fraction could reduce the effective dosage of the chemotherapeutic agent, mitomycin-C (MMC) by increasing the proliferation, differentiation and activation of immunocompetent cells. It could also reduce the immunosuppressive activity caused by MMC [80].

Apart from the D-fraction, other polysaccharide fractions with immunomodulatory activity have also been isolated from *G. frondosa*. Ishibashi and coworkers isolated insoluble and a high-molecular-weight soluble forms of Grifolan (GRN) from *G. frondosa*, both of which can activate macrophages through triggering cytokine secretion to produce TNF [61,100]. Similarly, Mao et al. observed increased levels of TNF-α, IL-2, IL-1β and nitric oxide (NO) in the serum with the dosage of polysaccharide GP11 from *G. frondosa*, suggesting the activation of macrophages and the stimulation of tumoricidal activity [64]. Masuda and coworkers found that the anti-cancer activity of the polysaccharide fraction MZF from *G. frondosa* was associated with the activation of cell-mediated immunity resulting from the induction of macrophage proliferation, increasing levels of IL12, IL2, IFN-γ and TNF-α, as well as enhancement of NK cells and cytotoxic T lymphocytes [25,59]. The GFP fraction isolated by Meng et al. promoted the production of cytokines and chemokines such as IL-6, IFN-γ and TNF-α, and also effectively enhanced the proliferative activity of fibroblasts, contributing to strong immune-stimulating activity [68]. Table 7 presents the common testing methods and potency indices of immunomodulatory effects. In vitro testing is usually performed with cytokine production evaluated based on ELISA and macrophage activity by MTT assay.

### 4.3. Antiviral and Antibacterial Effects

There have been a number of studies reporting the beneficial effects of *G. frondosa* in the treatment of viral infections, including those caused by hepatitis B virus (HBV), enterovirus 71 (EV71), herpes simplex virus type 1 (HSV-1) and human immunodeficiency virus (HIV). Mayell and coworkers reported a study on patients with chronic hepatitis B. The results showed that patients who took *G. frondosa* fruiting body polysaccharides showed positive signs, specifically a higher recovery rate compared with the control group [1]. Nanba et al. reported that the MD-fraction from *G. frondosa* could fight against HIV through several pathways, including direct inhibition of HIV, stimulation of the natural defense system against HIV and a reduction in vulnerability to opportunistic infections [50]. The GFP1 fraction extracted by Zhao and coworkers was found to fight against EV71, the causative pathogen of hand-foot-and-mouth disease. The researchers found that *G. frondosa* could hinder EV71 viral replication, suppressing genomic RNA synthesis and protein expression, and thus could be used as a promising therapeutic compound for EV71 treatment [27]. In addition to polysaccharide fractions, the protein fraction GFAHP purified from *G. frondosa* by Gu et al., has also shown anti-viral effects. It significantly inhibited HSV-1 replication in vitro and reduced HSV-1 induced symptoms such as blepharitis in a murine model [35].

In addition to antiviral effect, the D-fraction isolated from *G. frondosa* has also shown antibacterial effects. Kodama and coworkers found that the mechanism of antibacterial action of D-fraction was related to the immune-stimulating activity. The D-fraction could activate immuno-competent cells and induce the production of cytokines, which further lead to the activity enhancement of splenic T cells to kill *Listeria monocytogenes* [84]. Unlike the antibacterial mechanism, the antiviral action of the D-fraction is not directly related to the immune system. According to Gu et al., the D-fraction interfered with HBV replication through the inhibition of HBV polymerase [85].

### 4.4. Antidiabetic Activity

The hypoglycemic effects of *G. frondosa* extracts have been demonstrated in multiple animal studies. To test the antidiabetic activity of active ingredients in *G. frondosa*, in vivo fasting serum glucose (FSG) or fasting blood glucose (FBG) measurements are generally performed after feeding the bioactive ingredients to animal models for 2 to 4 weeks (Table 7). A high FSG level is one of the characteristics of diabetes mellitus sufferers and the influence on FSG level could directly indicate the antidiabetic effect of active ingredients in *G. frondosa*.

The hypoglycemic mechanisms of these polysaccharide fractions are most likely to be linked to insulin activity. For instance, F2 and F3 polysaccharides and SX glycoprotein fractions have been suggested to exert hypoglycemic effect through insulin signal pathway [63,87]. Konno et al. reported that the SX-fraction could facilitate glucose uptake, leading to activation of the insulin receptor (IR) and insulin receptor substrate 1 (IRS-1), and eventually resulting in increased insulin secretion. In a normal situation, a high glucose level would lead to low glucose uptake, but the SX-fraction overcame this suppressive effect and impaired the insulin signaling pathway [87]. The hypoglycemic mechanisms of F2 and F3 polysaccharides were also related to IR and IRS-1. Xiao et al. reported that they could improve insulin sensitivity and decrease FSG levels by increasing protein levels of IR and decreasing protein levels of IRS-1 [63]. The anti-diabetic effects of MT-α-glucan, such as ameliorating insulin resistance of peripheral target tissue and improving insulin sensitivity, were also reported to be associated with IR [26].

In addition to the enhancement of insulin activity, the hypoglycemic effects of *G. frondosa* may be generated through the inhibition of α-glucosidase activity, because an anti-α-glucosidase effect could prevent starch hydrolysis into disaccharides and decrease the blood glucose level. Shen and coworkers examined the hyperglycemic effects of non-polar fractions in *G. frondosa* both in vivo and in vitro. Research findings showed that *G. frondosa* exhibited strong anti-α-glucosidase activity in vitro, and could significantly lower the blood glucose level in high-fat-diet-fed and streptozotocin-induced hyperglycemic animals [86]. Chen et al. attributed the anti-α-glucosidase effect to the pyrrole alkaloids and ergosterols extracted from *G. frondosa* [37], whereas Wu et al. suggested that the ergosterol peroxide isolated from *G. frondosa* contributed to its anti-diabetic effect [75]. However, Su and coworkers concluded that the strong anti-α-glucosidase activity was mainly attributed to the oleic acid and linoleic acid, rather than ergosterol and ergosterol peroxide in *G. frondosa* [101]. Some previous studies suggested that the anti-diabetic activity of *G. frondosa* originated from its regulatory effect on gut microbiota, which shall be discussed later in this paper.

### 4.5. Lipid Metabolism Regulation and Anti-Hypertension Effects

The effects of *G. frondosa* on lipid metabolism regulation and anti-hypertension have been shown in many reports. Kubo and Nanba found that with the *G. frondosa* fruiting body as the feed, the triglyceride, cholesterol and phospholipid levels in the serum of rats were suppressed by 30–80% compared with those of the control group of animals. Meanwhile, the weight of the extirpated liver was also 60–70% lower than that of the control group, and the corresponding cholesterol excretion ratio in feces increased by 1.8 times with *G. frondosa* treatment, further demonstrating that *G. frondosa* treatment helped improve lipid metabolism and inhibit increases in liver lipid and serum lipid after the ingestion of high-fat feed [88]. Similar results were obtained by Fukushima and coworkers, who showed that serum total cholesterol concentrations and very-low-density lipoprotein (VLDL) levels in rats fed with 50 g/kg *G. frondosa* were lowered compared with those of the control group (50 g/kg cellulose powder), and the fecal cholesterol excretion was significantly higher compared with the control group [89].

The antihypertensive effects of the active ingredients of *G. frondosa* have been determined mainly through the measurement of systolic blood pressure (SBP) in animal models, as summarized in Table 7. Kabir et al. conducted an experiment on hypertensive rats with a diet containing 5% *G. frondosa* or *Lentinus edodes* (*L. edodes*) mushroom powder. The results showed that both *G. frondosa* and *L. edodes* treatment could result in a significant decrease in the SBP of spontaneously hypertensive rats. The reduction in SBP level was similar for *G. frodosa* and *L. edodes*, which was around 15 mmHg after 63 days of a mushroom diet compared with the control group [102]. Their further research showed that *G. frondosa* could not only suppress the development of hypertension (preventive effect), but also lower elevated blood pressure (treatment effect) [103]. Preuss and coworkers compared rats fed with two commercially-available fractions of SX and D with a control group fed on a baseline diet and found that *G. frondosa* fractions could lessen age-related hypertension partly via their effects on the renin-angiotensin system [90].

### 4.6. Antioxidant Activities

Several components in *G. frondosa*, including polysaccharides, proteins, fatty acids and some other constituents, have shown notable antioxidant activities. The common activity assay methods and anti-oxidant potencies are summarized in Table 7. As shown in this table, the common antioxidant activity assays include the scavenging abilities of hydroxyl radicals, DPPH radicals, superoxide radicals and hydrogen peroxide; as well as the reducing power and Fe^2+^ chelating activity. Lee and coworkers suggested that polysaccharides from *G. frondosa* could be potential ingredients for cosmetic applications due to their antioxidant activity, radical scavenging activity after UV irradiation, proliferation of fibroblasts and collagen biosynthesis [92]. Similar findings were obtained by Chen and coworkers, who showed that the crude polysaccharide GFP, extracted from *G. frondosa* fruiting bodies, possessed significant inhibitory effects on hydroxyl, superoxide and DPPH radicals [93].

The antioxidant activity of *G. frondosa* polysaccharides could be further enhanced by incorporation of zinc or selenium. Zhang and coworkers used the strain of *G. frondosa* as a vector of zinc biotransformation to produce zinc-incorporated intracellular polysaccharides, which showed notable antioxidant and anti-aging activities compared with the corresponding non-zinc-incorporated intracellular polysaccharides [91]. Li and coworkers purified crude Se-polysaccharides (Se-GFP) from the fruiting bodies of Se-enriched *G. frondosa* and obtained a heteropolysaccaride of Se-GFP-22 with more remarkable antioxidant effects than that of non-Se-incorporated GFP-22. The antioxidant activity might be affected by the degree of branching, molecular weight and configuration, as well as the synergistic effect of polysaccharide and Se [67].

Other than polysaccharides, proteins, fatty acids and other molecules from *G. frondosa* such as phenols and flavonoids also showed antioxidant activities. Dong and coworkers hydrolyzed protein from the *G. frondosa* fruiting body using different proteases and found that trypsin hydrolysate had the strongest antioxidant potential, especially the GFHT-4 fraction, with a molecular weight lower than 3 kDa [94]. Moreover, according to Zhang et al., the inhibition levels of cyclooxygenase (COX)-1 enzyme and COX-2 enzyme activities by a fatty acid of *G. frondosa* were 98% and 99%, respectively. Moreover, the inhibition of liposome peroxidation by the fatty acid was also as high as 79% [73]. Yeh and coworkers obtained several antioxidant components including flavonoids, phenols, α-tocopherol and ascorbic acid from the ethanol, cold-water and hot-water extracts of *G. frondosa*. All of these extracts exhibited various antioxidant activities, including reducing power, chelating ferrous ions and scavenging DPPH and superoxide anions [39].

### 4.7. Gut Microbiota Regulation

In recent years, there has been growing evidence of the important role of gut microbiota in the mediation/action of the various health benefits of mushrooms, especially their polysaccharide components [104]. Many studies have investigated the regulation of gut microbiota by the bioactive polysaccharides from edible and medicinal mushrooms such as *G. frondosa* because the biological macromolecules of polysaccharides, which cannot be directly absorbed, can be utilized by intestinal flora [105]. Friedman reviewed mushroom polysaccharides and their anti-obesity, anti-diabetes, anti-cancer and antibiotic properties, and suggested that the regulation of gut microbiota by polysaccharides was the major mechanism behind these properties [106]. Specifically, the maintaining of gut microbiota homeostasis has been found to be related with improved treatment of type 2 diabetes mellitus (T2DM) [107] and non-alcoholic fatty liver disease (NAFLD) [108]. To evaluate the microbiota regulation activity of bioactive components, in vivo measurement of gut microbiota is usually preferred by high throughput sequencing, as shown in Table 7.

Very recently, Chen and coworkers reported the regulatory efficacy of a novel *G. frondosa* polysaccharide GFP-N on the intestinal microflora of diabetic groups in vivo using single-molecule real-time sequencing technology (SMRT) [31]. There were significant differences exhibited in the composition of microbial populations in gut microbiota between the GFP-N-treated group and the diabetic control group. The relative abundance of some bacterial species such as *Lactobacillus acidophilus* (*L. acidophilus*) and *Bacteroides acidifaciens* (*B. acidifaciens*) was increased with GFP-N treatment. *L. acidophilus* has been shown to delay the progression of high fructose-induced diabetes in rats [96], and *B. acidifaciens* has shown the potential for treatment of metabolic diseases such as obesity and diabetes [109]. Guo et al. obtained similar results, showing that GFP could regulate intestinal microflora by significantly elevating the relative abundance of *Alistipes* and *Bacteroides* and reducing *Enterococcus*, which was associated with the improved hyperlipidemia and hyperglycemia in T2DM induced by streptozotocin and a high-fat diet (HFD) [107]. The same research group also developed *G. frondosa* polysaccharide-chromium (III) (GFP-Cr(III)) through chelation because chromium (III) was the most important trace mineral for T2DM treatment. Compared with inorganic chromium, organic chromium (III) has been found to have much better effects, with lower toxicity and genotoxicity. The researchers found that GFP-Cr(III) not only had the effects of GFP as shown in their previous work but also significantly increased the relative abundance of *Enterorhabdus* and *Coriobacteriaceae* due to the presence of Cr(III) [110].

Additionally, GFP has also been found to regulate the gut microbiota of rats with non-alcoholic fatty liver disease (NAFLD). Li and coworkers found that GFP could partly recover the HFD-induced alteration of cecal microbiota structure [30]. GFP treatment could decrease the *Firmicutes* to *Bacteroidetes* ratio, indicating a lower possibility of lipid production from undigested carbohydrates [108]. Friedman also suggested in his review that the ratio decrease of the two major classes of gut bacteria, namely, *Firmicutes* and *Bacteroides*, could have fat-lowering effects in obesity treatment [106]. In addition, GFP supplementation significantly increased the proportion of *Allobaculum*, *Bacteroides*, *Bifidobacterium* and some other microbial groups in the cecal microbiota, which might boost the immune system of the host and the defense against NAFLD [30]. The boosting of the immune system may contribute to the anti-tumor and anti-inflammatory effect of GFP as well [29,111]. Nevertheless, the functions of gut microbiota on the various bioactivities of *G. frondosa* polysaccharides and other components still require further exploration.

## 5. Conclusions

Edible and medicinal fungi or mushrooms are among the most common sources of health foods and nutraceutical products. *G. frondosa* is one of the most widely explored fungal species for nutraceutical and therapeutic compounds. The fungal biomass of *G. frondosa* displays a high content of proteins and carbohydrates and a relatively low content of fat compared with other commonly cultivated mushrooms. The crude water extracts, isolated fractions and purified components have shown a number of bioactivities, including antitumor, immunomodulatory, antiviral, antibacterial, antidiabetic, lipid metabolism regulation, hypertension control and antioxidation. Some of these health effects may be associated with the regulation of the human gut microbiota. Polysaccharides, which represent the most significant bioactive components of *G. frondosa*, contribute to many of its bioactivities and health benefits. The most successful and valuable health products from this fungal species are represented by the polysaccharide fractions and polysaccharide protein complexes, including the D-fraction or the MD-fraction and Grifolan, which have been approved for human use in immunotherapy and complimentary treatment of cancers with chemotherapy and radiotherapy.

Although some of the constituents of *G. frondosa* have been widely used in health foods or dietary supplements, very few have been used in prescribed medication, which requires more rigorous assessment and clinical trials. For a wider and more reliable application of the various components in nutraceutical and therapeutic products, it is fundamental to gain a better understanding of the structure–bioactivity relationship and the underlying mechanisms of action in the human body. Structural modification of the polysaccharides is another feasible strategy to attain enhanced bioactivity and novel bioactive molecules. As for many food and medicinal products, good manufacturing practice (GMP) should be implemented in the production process and systems, and standardized protocols should be established and followed for the preparation and quality control of the useful components. With increasing public concern about health threats from food contamination, environmental pollution and new infectious organisms such as the COVID-19 virus, the protection of human health through the immunomodulatory and health-promoting functions of *G. frondosa* constituents is even more attractive and promising. Therefore, it is worthwhile to put more effort into the research and development of this edible fungal species.

## Figures and Tables

**Table 1:** Proximate composition of *G. frondosa*’s fruiting body and mycelium.

Components ^1^ (%)	Fruiting Body								Mycelium			
	[8] *	[9] ^#^	[12] ^#^	[10] ^2,#^	[13] ^#^	[11] ^2,#^	[7] ^2,#^	Average	[43]	[44] ^2^	[11] ^2^	Average
Moisture	83.1	89.1	90.9	86.1	90.4	95.6	95.2	90.1 ± 4.5	84.8	96.7	92.3	91.3 ± 6.0
Dry matter ^3^	16.9	10.9	9.1	13.9	9.6	4.4	4.8	9.9 ± 4.5	15.2	3.3	7.7	8.7 ± 6.0
Carbohydrate ^4^	70.4	74.9	72.3	68.8	71.8	66.3	70.3	70.7 ± 2.7	66.3	45.0	60.4	57.2 ± 11.0
Crude ash	6.5	4.8	6.6	7.0	7.1	6.2	4.9	6.1 ± 0.9	6.4	4.0	4.7	5.0 ± 1.3
Crude fat	4.5	1.5	3.3	3.1	2.4	6.5	5.6	3.8 ± 1.8	4.2	24.7	6.5	11.8 ± 11.2
Crude protein	18.6	18.9	17.8	21.1	18.8	21.0	19.2	19.3 ± 1.3	23.1	26.4	28.4	26.0 ± 2.7

^1^ Moisture and dry matter were based on fresh weight; others were presented based on dry weight. ^2^ For easy comparison, only the mean value is used. ^3^ The dry matter was represented by (1 − moisture/total) × 100%. ^4^ Amount of carbohydrate was calculated by subtracting crude ash, crude fat and crude protein. * Fruiting body was grown naturally. ^#^ Fruiting body was grown artificially.

**Table 2:** Soluble sugar content of *G. frondosa* fruiting body and mycelium in dry weight.

Component	Fruiting Body (mg/g Dry wt.)			Mycelium (mg/g Dry wt.)		
	[9] ^#^	[10] ^1,#^	[11] ^1,#^	[43]	[44] ^1^	[11] ^1^
Arabinose	n.d. ^2^	n.d.	n.d.	n.d.	n.d.	5.37
Arabitol	n.d.	n.d.	n.d.	n.d.	12.65	2.01
Fructose	n.d.	n.d.	n.d.	1.00	n.d.	2.99
Glucose	59.30	14.02	2.42	8.00	19.72	2.18
Lactose	n.d.	n.d.	n.d.	n.d.	n.d.	0.93
Mannitol	7.20	9.36	1.00	n.d.	9.92	2.30
Mannose	n.d.	n.d.	n.d.	n.d.	n.d.	1.92
Ribose	n.d.	n.d.	8.34	n.d.	n.d.	4.04
Trehalose	45.80	161.83	99.94	65.00	41.60	65.32
Total	112.30	185.21	111.7	74.00	83.89	87.06

^1^ For easy comparison, only the mean value is used. ^2^ Not determined or not detected. ^#^ Fruiting body was grown artificially.

**Table 3:** Free amino acid assay of *G. frondosa*’s fruiting body and mycelium in dry weight.

Component (mg/g Dry wt.)	Fruiting Body				Mycelium	
	[13] ^1,#^		[11] ^1,#^	[10] ^1,#^	[44]	[11] ^1^
	In Sawdust	In Log				
L-Alanine	2.15	3.13	5.22	2.77	3.26	14.59
L-Arginine	3.02	3.21	1.66	0.64	0.97	12.39
L-Aspartic acid	1.61	1.25	1.88	0.42	2.75	19.40
L-Glutamic acid	8.01	9.10	12.62	0.67	3.76	2.10
GABA	n.d. ^2^	n.d.	0.28	n.d.	n.d.	17.09
Glycine	1.53	1.53	2.46	0.57	1.93	7.81
L-Histidine ^3^	1.53	0.94	19.50	0.59	4.10	n.d.
L-Isoleucine ^3^	0.12	0.12	0.56	0.33	2.80	6.67
L-Leucine ^3^	0.05	0.09	0.27	0.35	4.92	6.39
L-Lysine ^3^	1.56	1.28	5.70	1.11	0.22	23.49
L-Methionine ^3^	n.d.	n.d.	4.50	1.40	0.67	n.d.
L-Phenylalanine ^3^	0.26	0.28	2.71	0.80	1.66	9.98
L-Serine	2.91	2.82	2.01	0.97	2.73	10.74
L-Threonine ^3^	1.43	1.44	n.d.	4.40	8.23	10.85
L-Tryptophan ^3^	n.d.	n.d.	n.d.	0.27	n.d.	12.01
L-Tyrosine	1.77	0.73	1.53	n.d.	2.15	17.99
L-Valine ^3^	0.96	0.91	0.39	0.60	4.13	9.41
Total	29.26	29.38	61.29	15.9	44.28	180.91

^1^ For easy comparison, only the mean value is presented. ^2^ Not determined or not detected. ^3^ Essential amino acid. ^#^ Fruiting body was grown artificially.

**Table 4:** Bioactive polysaccharide fractions isolated from *G. frondosa* with significant medicinal values.

Name of Active Fractions/ Purified PS	MW	Structure/Composition	Monosaccharide Composition *	Extraction Solvent & Source	Reference
Grifolan-7N	1200 kDa	(1→3)-linked β-D-glucan having a single β-D-glucopyranosyl group attached to position 6 of almost every 3rd backbone unit	Glc	Hot sodium hydroxide, Fruiting body	[18]
GRN	500 kDa (Mw)	(1→6) –branched (1→3)-β-D-Glucan	Glc	0.5% citrate buffer, Mycelium	[24]
X-fraction	-	β-1,6 glucan having alpha-1,4 branches	Glc	EtOEt-EtOH and then hot water, Fruiting body	[23]
D-fraction	1000 kDa	Isolated beta-glucan polysaccharide compounds (beta-1,6 glucan and beta-1,3 glucan) with protein	Glc	Hot water, Fruiting body	[22,50]
MD-fraction	1000 kDa	Purified D-fraction with the same main component where the glucan/protein ratio is in the range of 80:20 to 99:1	Glc	Hot water, Fruiting body	[22]
MZ-fraction	20 kDa (Mw)	β-1,6 main chain and a β-1,3 side chain	Glc	Hot water, Fruiting body	[25]
GFPS1b	21 kDa	Backbone consisted of a-(1→4)-linked D-galacopyranosyl and a-(1→3)-linked D-glucopyranosyl residues substituted at O-6 with glycosyl residues composed of a-L-arabinose-(1→4)-a-D-glucose (1→linked residues	Glc: Gal: Ara = 4:2:1	Hot water, Mycelium	[57]
EX-GF-Fr. III	2.8 kDa	-	Glc: Rib: Man: Gal: Rha: Xylose = 3.98:1.44:1.34:1.00:0.41:0.15	Mycelium	[58]
MZF	23 kDa	→6)-α-d-Galp-(1→(36.2%),→3)-α-l-Fucp-(1→(14.5%),→6)-α-d-Manp-(1→(9.4%),→3)-β-d-Glcp-(1→(10.1%), α-d-Manp-(1→(23.2%), and →3,6)-β-d-Glcp-(1→(6.5%)	Gal: Man: Fuc: Glc = 1.24:1:0.95:0.88	Hot water, Fruiting body	[59]
GFPBW1	300 kDa	β-D-(1-3)-linked glucan backbone with a single β-D-(1-6)-linked glucopyranosyl residue branched at C-6 on every third residue	Glc	Hot water then 5% NaOH solution, Fruiting body	[60]
GFPBW2	26.2 kDa	Backbone consisting of β-D-1,3- and β-D-1,4-linked glucopyranosyl residues, with branches attached to O-6 of β-D-1,3-linked glucopyranosyl residues	Glc	Hot water then 5% NaOH solution, Fruiting body	[61]
MT-α-glucan	40–45 kDa	D-glucose with α-glucosidic bond	Glc	Hot water, fruiting body	[26,52,53]
GFPW	15.7 kDa	Backbone of α-1,6-linked galactopyranosyl residues with branches attached to O-2 of α-1,3-linked fucose residues and terminal mannose	Man: Fuc: Gal = 0.41:0.44:1	Hot water, fruiting body	[62]
GFPs-F2 and F3	-	F2 with polysaccharide 62.5% and protein 37.5%; F3 with polysaccharide 78.3% and protein 21.7%	F2: Glc: Man: Gal: Xyl: Ara: Rha: Rib = 26.74:22.79:16.76: 16.02:14.29:2.05:1.35. F3: Ri: Ara: Xyl = 74.74:14.20:11.08	Hot water, fruiting body	[63]
GP11	6.9 kDa	→1)-d-Manp-(6→,→1)-d-Glcp-(4→,→1)-d-Galp-(6→and→2,3,6)-d-Glcp-(1→, with branches attached at O-2,3 of 1,2,3,6-linked Glcp residues and terminal T-Glcp	Man: Glc: Gal = 1:5.04:2.61	Hot water, fruiting body	[64]
GRP1	40.5 kDa	1,6-β-D-glucan backbone with a single1,3-α-D-fucopyranosyl side-branching unit	Glc: Fuc = 2.3:0.5.	Hot water, mycelium	[27]
GFP-A	848 kDa	Main chain consisted of (1→4)-linked and (1→6)-linked α-D-glucopyranosyl, and (1→3,6)-linked α-D-mannopyranosyl residues	Rha: Ara: Xyl: Gal: Man: Glc = 1.38:0.53:0.11:1.07:28.75:1.76	Hot water, fruiting body	[54,65]
GFP-A	2484 kDa	α-type rhamnopyranose, β-type mannopyranose and α-type galactopyranose	Rha: Xyl: Man: Glc: Gal = 25.98: 9.32: 11.73: 4.74: 48.22	Ultrasound and hot water, fruiting body	[55]
Se-GP11	33 kDa	-	Man: Glc: Gal = 1:4.91:2.41	Hot water, fruiting body	[66]
Se-GFP-22	4130 kDa	Backbone chain of 1,4-α-D-Glc*p* units with a branched point at C6 of both 1,3,6-β-D-Man*p* and 1,4,6-α-D-Gal*p* units	Man: Glc: Gal = 3.3:23.3:1	Hot water, fruiting body	[67]
GFP	155 kDa	(1→4)-linked methylation backbone, Glcp residues were major structural polysaccharide GFP units, accounting of the polysaccharide backbone speculate GFP every 3)-Glcp-(1→and one 3,4)-Glcp-(1→connected interval with a small amount of 1→, 1→4,1→6 glycosidic linkage	Rha: Xyl: Man: Glc = 1.00:1.04:1.11:6.21	Hot water, fruiting body	[68]
GFP30-2-a	2040 kDa	Repeating unit of β D Glcp (1→[4) α D Glcp (1→4) α D Glcp (1]_m_→4) α D Glcp	Glc: Gal = 1:0.098	Hot water, fruiting body	[69]
GFP-22	27.2 kDa	Backbone composed of 1,4-β-D-Glcp, 1,3-β-D-Glcp, 1,6-α-D-Glcp, 1,6-α-D-Galp, 1,4,6-α-D-Manp and 1,3,6-α-D-Manp units	Man: Glc: Gal = 2.8:15.2:1.0	Hot water, fruiting body	[70]
GF70-F1	1260 kDa	(1→3),(1→6)-β-D-glucan &β-(1→4)- linked backbone and β-(1→6)-linked branches	Gal:Glc:Man = 1.24:56:1	Hot water, fruiting body	[28]
LMw-GFP	1790 Da	α-T-Glcp (28.26%), α-1→4-Glcp (50.24%) and α-1→3,4-Glcp (21.50%)	Glc	65 °C water with ultrasound, fruiting body	[51]
GFAP	644.9 kDa	(1→3)-β-D-Glcp and (1→3)-α-D-Manp	Gal: Glc:Man = 0.23:2.18:1	Water with ultrasound, fruiting body	[56]
GFP-N	1.26 × 10^7^ Da	→2,6)-α-D-Manp-(1→4, α-L-Araf-C1→ and →3,6)-β-DGlcp-(1→	Ara: Man: Glc = 3.79:1.00:49.70	Hot water, fruiting body	[31]

* Ara: Arabinose; Fuc: Fucose; Gal: Galactose; Glc: Glucose; Man: Mannose; Rha: Rhamnose; Rib: Ribose; Xyl: Xylose.

**Table 5:** Bioactive proteins and peptides isolated from *G. frondosa* with medicinal values.

Bioactive Protein/Peptide	MW	Composition *^,#^/Structure	Extraction Solvent & Source	Ref.
GFL	30–52 kDa	Glycoprotein with 3.3% total sugar, amino acids with a high content of acidic and hydroxyl amino acids and a low content of Met and His	2-Mercaptoethanol and Ethylenediaminetetraacetic acid (EDTA), fruiting body	[71]
Glyco-protein	20 kDa	Protein to saccharide ratio from 75:25 to 90:10. Amino acid composition: Asn, Gln, Ser, Thr, Gly, Ala, Val, Cys, Met, Ile, Leu, Tyr, Phe, Lys. His, Arg and Pro. Monosaccharide composition: Gal, Man, Glc, N-acetylglucosamine and Fuc	Ethanol then hot water, fruiting body	[34]
GFAHP	29.5 kDa	N-terminal sequence consisted of an 11-amino-acid peptide.	Hot water, fruiting body	[35]
GFG-3a	88.01 kDa	Glycoprotein with O-glycosylation and 6.20% carbohydrate composed of Ara, Fru, Man and Glc in a molar ratio of 1.33:4.51:2.46:1.00; predominantly β-sheet glycoprotein with a relatively small α-helical content	Water, mycelium	[32]
GFPr	83 kDa	Non-glucan heterodimeric protein that consists of two 41 kDa subunits	Buffer containing acetic acid, 2-mercaptoethanol, and sodium chloride, fruiting body	[33]

* Ala: alanine; Arg: arginine; Asn: asparagine; Cys: cysteine; Gln: glutamine; His: histidine; Gly: glycine; Ile: isoleucine; Leu: leucine; Lys: lysine; Met: methionine; Phe: phenylalanine; Pro: proline; Ser: serine; Thr: threonine; Tyr: tyrosine; Val: valine. ^#^ Ara: arabinose; Fru: fructose; Gal: galactose; Glc: glucose; Man: mannose; Fuc: fucose.

**Table 6:** Bioactive small molecules isolated from *G. frondosa*.

Name of Molecule/Fractions	Composition	Extraction Solvent & Source	Ref.
Fatty acid, Compounds **1**,**2**,**3**	Fatty acid composed of as palmitic, oleic, and linoleic acids; ergosterol (**1**), ergostra-4,6,8(14),22-tetraen-3-one (**2**), 1-oleoyl-2-linoleoyl-3-palmitoylglycerol (**3**)	Hexane, mycelium	[73]
HE-5-5	*o*-orsellinaldehyde	Ethyl acetate, mycelium	[38]
Polyphenolics, flavonoids, ascorbic acid and α-tocopherol	-	Hot water/cold water/ethanol, fruiting body	[39]
AGF	-	Acetone, mycelium	[36]
Grifolaone A	(S)-methyl 2-(2-hydroxy-3,4-dimethyl-5-oxo-2,5-dihydrofuran-2-yl) acetate	Ethyl acetate, mycelium	[74]
GF-3	Pyrrolefronine, seven pyrrole alkaloids and nine ergosterols	Ethanol, fruiting body	[37]
Ergosterol peroxide	-	Methanol, fruiting body	[75]

**Table 7:** Bioactivity potency and testing methods of major bioactive molecules isolated from *G. frondosa*.

Bioactivity	Bioactive Components	Name of Fraction	Testing Method	Potency of Bioactivity	Ref.
Anti-tumor	PS	D-fraction	In vivo counting of the number of tumor foci metastasized using stereoscope wide field microscopy	1 mg/kg/day for 17 days against MM46 liver carcinoma, 91.3% inhibition ratio	[80]
	PS	GFP-A	In vitro testing of cancer cell viability using MTT assay	150 μg/mL at 48 h, 50% inhibition (IC_50_) of human colon cancer cells	[65]
	PS	MD-fraction	In vivo assessing of inhibition rate by measuring tumor weight	0.1 mg/kg at 10 times after transplanting MM46 carcinoma, 94.3% inhibition ratio	[22]
	PS	MZ-fraction	In vivo assessing of tumor inhibition by measuring tumor weight	4 mg/kg/day against MM46 carcinoma, 70.3% inhibition ratio	[25]
	PS	GFP-A	In vivo assessing of tumor inhibition rate in mice inoculated with S180 sarcoma cells	Oral administration of 50, 100 and 200 mg/kg for 15 days, tumor inhibitory rates were 17.1%, 28.3% and 52.2% respectively	[55]
	PS	LMw-GFP	In vivo assessing of tumor inhibition rate in mice inoculated with H22 hepatoma cells	Oral administration of 200 mg/kg for 15 days, tumor inhibitory ratio was 40.1%	[51]
	PS	GFAP	In vivo assessing of tumor inhibition rate in H22 hepatoma cell-bearing mice	Intragastric administration of 100 and 200 mg/kg for 15 days, tumor inhibitory rate was 16.36% and 36.72% respectively	[56]
	Glycoprotein	GFG-3a	In vitro testing of cancer cell viability using MTT assay	20 μg/mL against sarcoma 180 cells, 92% inhibition ratio; 60 μg/mL against BEL 7402 cells, 95% inhibition ratio	[32]
	Water soluble extract	-	In vitro counting under a phase-contrast microscope	10% *w/v* Maitake extract against TMK-1 gastric cancer cell lines for 3 days, 90% inhibition ratio	[81]
Immuno-modulatory	PS	D-fraction	In vitro evaluation of cytokine production using ELISA	4.0 mg/kg/day, 3000 pg/mL IL-12 production	[82]
	PS	GRN	In vitro evaluation of cytokine production and activity of macrophages using ELISA and MTT assay	100 μg/mL, 11.050 ng/mL IL-6 production and 14.458 ng/mL TNF-α production by RAW264.7 cells	[24]
	PS	GP11	In vitro evaluation of cytokine production and activity of macrophages using ELISA and MTT assay	1000 μg/mL, 81.84 pg/mL TNF-α, 229.07 pg/mL IL-1β level	[64]
	PS	MZ-fraction	In vitro determination of TNF-α or IL-12 by ELISA	500 μg/mL, around 85 pg/mL IL-12 and 50 ng/mL TNF-α by J774.1 macrophage	[25]
	PS	GFP	In vitro macrophage proliferation assessment using MTT assay; concentration of cytokine and chemokine measured by multiplex magnetic bead panel kit	40 μg/mL, 150% cell viability for 36 h	[68]
	PS	Fr. I and II	In vitro human blood cytokine concentrations determined by ELISA	0.1 mg/mL Fr. II, around 3700 pg/mL TNF-α, 360 pg/mL IFN-γ and 4400 pg/mL IL-6	[83]
Antiviral/ antibacterial	PS	D-fraction	In vivo determination of the survival rate of *Listeria monocytogenes* by estimating colony-forming units (CFUs); In vitro HBV DNA and viral antigen analysis using quantitative real-time polymerase chain reaction and end-point titration in radioimmunoassays, respectively	10 mg/kg/d, survived rate of *L. monocytogenes* = 67%; IC_50_ for HBV DNA in cells = 0.59 mg/mL; IC_50_ for HBV polymerase = 1.38 mg/mL;	[84,85]
	PS	GFP1	In vitro EV71-infected cell inhibition rate determination using CCK-8 assay	250 μg/mL extract, inhibition rate = 20% after 10 h	[27]
	Protein	GFAHP	In vitro HSV-1 virus quantity analysis using plaque reduction assay; In vivo HSV-1 virus measurement using plaque assay	IC_50_ for HSV-1 replication = 4.1 μg/mL; 150 μg/mL, mean virus titer 12.7% compared to control after 24 h	[35]
Antidiabetic	PS	F2/F3	In vivo fasting serum glucose (FSG) level measurement using glucose oxidase method in diabetes rat model	Intake 100 mg/kg/d F2 or 50 mg/kg/d F3 for two weeks, inhibits a rise in FSG level	[63]
	PS	MT-α-glucan	In vivo glucose oxidase method using reflective glucometer on KK-Ay mice	Intake 150 mg/kg/d for two weeks, decrease around 23% FSG	[26]
	n-hexane extract	GF-H	In vitro α-amylase inhibition assay and α-glucosidase inhibition assay; In vivo glucose level measurement by the glucose oxidase method in high-fat-diet and streptozotocin (HFD + STZ)-induced hyperglycemic mice	IC_50_ of α-amylase and α-glucosidase: 3.75 mg/mL and 0.04 mg/mL respectively; Intake 600 mg/kg, blood glucose level decrease 28%	[86]
	Glycoprotein	SX-fraction	In vivo FBG measurement on type 2 diabetic patients	Intake 2–4 weeks, 30–63% decline in FBG	[87]
	Small molecules	Ergosterol peroxide	In vitro assessment of antidiabetic activity in palmitate-induced murine C2C12 skeletal muscle cells by measuring glucose uptake	At 5 μM, the increase in the glucose absorption rate was as good as that of the insulin-treated cells	[75]
Lipid metabolism/ hypertension	Dry Maitake powder	-	In vitro testing using a commercial kit (cholesterol E-Test, Phospholipid B-Test Triglyceride E-Test)	Liver weight 0.68 times lower than control; Triglyceride, total cholesterol and free cholesterol reduced by 0.46 times, 0.54 times and 0.65 times in liver with diet containing 20% maitake for 11 d	[88]
	Dry Maitake fiber	-	In vitro total cholesterol, HDL cholesterol and triglyceride concentrations in the serum, determined enzymatically by commercially available reagent kits	Serum total cholesterol concentration reduced by 11% than control by 50 g/kg maitake for 4 weeks	[89]
	Water extract	-	In vivo systolic blood pressure (SBP) level measurement using tail plethysmography in aging female rats	Intake 350 mg/kg for 120 d, significantly lower SBP level	[90]
Antioxidant	PS	IZPS	In vitro access of hydroxyl radical, DPPH radical, superoxide radical and hydrogen peroxide scavenging ability, reducing power and Fe^2+^chelating activity by chemical methods	EC_50_ scavenging •OH, DPPH• and O^2−^ are 204 mg/L; 211 mg/L and 525 mg/L; At 1000 mg/L, H_2_O_2_ scavenging rate 95%; reducing power (abs at 700 nm) 0.38; Fe^2+^chelating activity 51%	[91]
	PS	G-2/G-3	In vitro assessment of the superoxide scavenging activity by chemical assay; In vitro assessment of free radical scavenging activity after UV irradiation in HDF cells	At 0.2% w/v, inhibit 90% (G2) and 75% (G3) O^2−^; decreased free radicals (formed after UV irradiation) by 20% for both G2 and G3	[92]
	PS	GFP-1, GFP-2/GFP-3	In vitro assessment of of hydroxyl radical, DPPH radical and superoxide radical scavenging ability and Fe^2+^chelating activity by chemical methods	At 3.0 mg/mL, the scavenging rate of DPPH•, •OH, O^2−^•: 49, 48 and 45% (GFP-1); 78, 53 & 53% (GFP-2) & 66, 93 & 83% (GFP-3); At 5 mg/L, Fe^2+^chelating rate: 91% (GFP-1); 98% (GFP-2) and 80% (GFP-3)	[93]
	PS	Se-GFP-22	In vitro assessment of DPPH radical scavenging ability	At 1000 μg/mL, 46% scavenging rate	[67]
	Protein	GFHT-4	In vitro assessment of DPPH radical scavenging ability, Fe^2+^chelating activity, reducing power and inhibition of linoleic acid autoxidation power by chemical methods	At 2.5 mg/mL, inhibits 90% DPPH•, chelate 80% Fe^2+^, reducing power close to 1.5 mg ascorbic acid/mL; At 0.5 mg/mL, inhibition of linoleic acid autoxidation power equivalent to BHA (0.5 mg/mL)	[94]
	Small molecule	Ergosterol, ergostra-4,6,8(14), 22-tetraen-3-one, & 1-oleoyl-2-linoleoyl-3-palmitoylglycerol	In vitro assessment of antioxidants by liposome oxidation model	At 100 μg/mL, 79, 48% and 42% inhibition rate, respectively	[73]
Microbiota regulation	PS	GFP	In vivo measurement of gut microbiota in high-fat-diet-fed rats by high-throughput sequencing	For GFP (400 mg/kg day)-treated group, significant increase in the relative abundance of *Helicobater*, *Intestinimonas*, *Barnesiella*, *Parasutterella*, *Ruminococcus* and *Flavonifracter*, and decrease in Clostridium-XVIII, *Butyricicoccus* and *Turicibacter*. Similar gut microbiota composition to that of the normal group.	[95]
	PS	GFP-N	In vivo determination of intestinal microflora in a diabetes rat model using single-molecule real-time sequencing	For GFP-N-fed group (75 and 150 mg/kg day), significant increase of the relative abundances of *Porphyromonas gingivalis*, *Akkermansia muciniphila*, *Lactobacillus acidophilus*, *Tannerella forsythia*, *Bacteroides acidifaciens* and *Roseburia intestinalis*. Similar gut microbiota composition to that of the normal group.	[31]
	PS	GFP	In vivo access of gut microbiota in high-fat diet-fed and streptozotocin-treated mice by high throughput sequencing.	For GFP-treated (900 mg/kg day) group, significant increase in the relative abundance of *Alistipes* and *Bacteroides*, and decrease in *Enterococcus*.	[96]
	PS	GFP	In vivo evaluation of gut microbiota in high-fat-diet-fed rats by high-throughput next-generation 16S rRNA gene sequencing.	For GFP-treated (150 mg/kg day) group, significant increase in the relative abundance of *Allobaculum*, *Bacteroides*, *Bifidobacterium* and other cecal microbiota compared with the HFD-fed group.	[30]
	PS	GFWE	In vivo access of gut microbiota in high-sucrose- and high-fat-diet-fed rats by real-time sequencing.	For GFWE-treated (150 mg/kg day) group, increase in the relative abundance of caecal bacteria *Oscillibacter* and *Barnesiella*.	[97]
	Small molecule	GF95 (mainly 4-hydroxyhippuric acid, flavone derivatives, luteolin, luteolin 6,7-dimethoxy & jaceosidin or 5,7,4-trihydroxy-3)	In vivo access of gut microbiota in high-fat-diet-fed rats by real-time sequencing	For GF95 (150 mg/kg day)-fed group, a higher relative abundance of *Intestinimonas* and *Butyricimonas* than that fed with HFD only.	[98]

## Data Availability

This review study used published data in the reference literatures.

## Abbreviations

*B. acidifaciens*:	*Bacteroides acidifaciens*
EV71:	enterovirus 71
FBG:	fasting blood glucose
FSG:	fasting serum glucose
*G. frondosa*:	*Grifola frondosa*
GFP:	*Grifola frondosa* polysaccharide
GRN:	Grifolan
HBV:	hepatitis B virus
HFD:	high-fat diet
HIV:	human immunodeficiency virus
IFN:	interferons
IL:	interleukins
IR:	insulin receptor
IRS-1:	insulin receptor substrate 1
HSV:	herpes simplex virus
*L. acidophilus*:	*Lactobacillus acidophilus*
*L. edodes*:	*Lentinus edodes*
NAFLD:	non-alcoholic fatty liver disease
NK cell:	natural killer cell
SBP:	systolic blood pressure
SSF:	solid-state fermentation
T2DM:	type 2 diabetes mellitus
TNF:	tumor necrosis factors
